# The first complete mitochondrial genome of *Siphonosoma* from *Siphonosoma* cumanense (Sipuncula, Sipunculidae)

**DOI:** 10.1080/23802359.2020.1721364

**Published:** 2020-01-31

**Authors:** Shengping Zhong, Lianghua Huang, Yonghong Liu, Guoqiang Huang, Xiuli Chen

**Affiliations:** a Institute of Marine Drugs, Guangxi University of Chinese Medicine, Nanning, China;; b Key Laboratory of Marine Biotechnology, Guangxi Institute of Oceanology, Beihai, China;; c Guangxi Key Laboratory of Aquatic Genetic Breeding and Healthy Aquaculture, Guangxi Academy of Fishery Sciences, Nanning, China

**Keywords:** Mitochondrial genome, *Siphonosoma cumanense*, Sipuncula

## Abstract

*Siphonosoma cumanense* is economic important species in the fishery of southeast China. However, the current classification and the phylogeny of genus *Siphonosoma* had not been verified yet. Here, we report the complete mitochondrial genome sequence of *S. Siphonosoma*. The mitogenome has 15,917 base pairs and made up of total of 38 genes (13 protein-coding, 23 transfer RNAs and 2 ribosomal RNAs), and a putative control region. This study was the first available complete mitogenomes of *Siphonosoma* and will provide useful genetic information for future phylogenetic and taxonomic classification of Sipuncula.

Sipuncula (known as peanut worms) is an ancient group of exclusively marine worms which contain about 150 exclusively species with a global distribution (Lemer et al. 2015). The evolutionary and phylogenetic classifications of Sipuncula have long been contentious and the systematics of different taxonomic levels of Sipuncula has been revised recently(Kawauchi et al. 2012). *Siphonosoma cumanense* is an important fisheries resource for its valuable nutrition and valuable ingredients of chines traditional medicines. However, the current classification and the phylogeny of genus *Siphonosoma* has not yet been determined. Mitochondrial genome is useful molecular techniques for phylogenetic relationships and taxonomy identification, especially when there is small number of unambiguous taxonomic characters available for taxonomy determination. Here, we report the first complete mitochondrial genome sequence of *Siphonosoma*, which will provide a better insight into phylogenetic assessment and taxonomic classification of Sipuncula.

The tissue samples of *S. Siphonosoma* from three individuals was collected from Beibu Bay, China (Beihai, 21.523154 N, 109.568641 E), and the whole body specimens (#GG0241) were deposited at Marine biological Herbarium, Guangxi Institute of Oceanology, Beihai, China. The total genomic DNA was extracted from the muscle of the specimens using an SQ Tissue DNA Kit (OMEGA, Guangzhou, China) following the manufacturer’s protocol. DNA libraries (350 bp insert) were constructed with the TruSeq NanoTM kit (Illumina, San Diego, CA) and were sequenced (2 × 150 bp paired-end) using HiSeq platform at Novogene Company, China. Mitogenome assembly was performed by MITObim (Hahn et al. 2013). The complete mitogenome of *Sipunculus nudus* (GenBank accession number: NC_011826) was chosen as the initial reference sequence for MITObim assembly. Gene annotation was performed by MITOS (http://mitos2.bioinf.uni-leipzig.de/).

The complete mitogenome of *S. Siphonosoma* from Beibu Bay was found to be 15,917 bp in length (GenBank accession number: MN813483), including the usual set of gene for mitogenome except for extra threonine tRNA which consisting of 13 protein-coding, 23 tRNA and 2 rRNA genes, and a putative control region. The overall base composition of the mitogenome was estimated to be A 27.5%, T 33.2%, C 25.4% and G 13.9%, with a high A + T content of 60.8%, which is slightly higher than *Sipunculus nudus* (57.5%) (Zhong et al. 2018), but is slightly lower than *Phascolosoma esculenta* (65.6%) (Zhao et al. 2019). The mitogenomic phylogenetic analyses showed that *S. Siphonosoma* was first clustered with genus *Sipunculus* and then clustered with genus *Phascolosoma* (Figure 1), which is consistent with the phylogenetic analyses of Sipuncula using four gene regions and morphology (Schulze et al. 2007). In the study of Sipunculan phylogeny based on six genes, however, the genus *Siphonosoma* had been revised within the new family Siphonosomatidae (Kawauchi et al. 2012). The taxonomic position of *Siphonosoma* need further study. Our study suggested closely related taxa between genus *Siphonosoma* and *Sipunculus*, which will contribute to further phylogenetic and taxonomic study of sipunculan worms.

**Figure 1.**
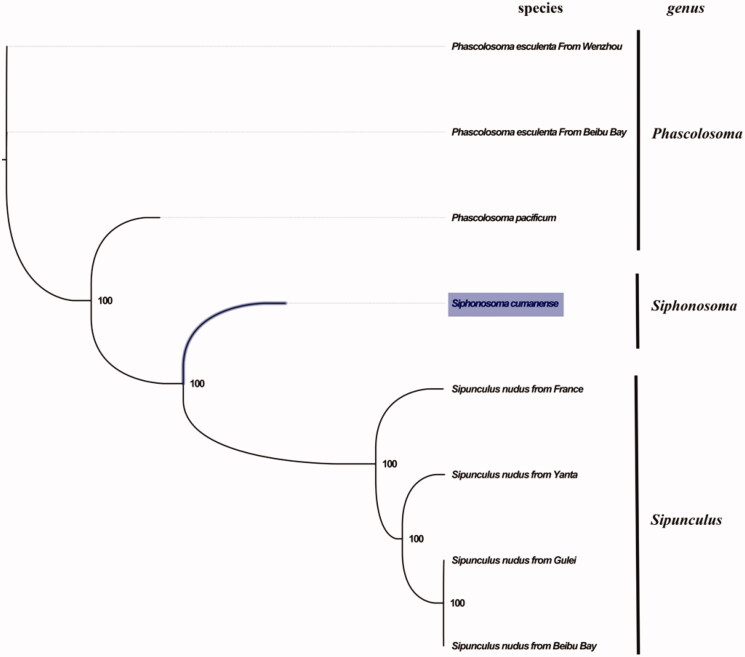
Phylogenetic tree in Sipuncula. The complete mitogenomes is downloaded from GenBank and the phylogenic tree is constructed by maximum-likelihood method with 100 bootstrap replicates. The bootstrap values were labeled at each branch nodes. The gene’s accession number for tree construction is listed as follows: *Phascolosoma pacificum* (NC_031412), *Phascolosoma esculenta* From Wenzhou (NC_012618), *Phascolosoma esculenta* From Beibu Bay (MG873458), *Sipunculus nudus* from France (NC_011826), *Sipunculus nudus* from Yanta (KP751904), *Sipunculus nudus* from Gulei (KJ754934), and *Sipunculus nudus* from Beibu Bay (MG873457).
